# Artificial Intelligence-Assisted Chest X-ray for the Diagnosis of COVID-19: A Systematic Review and Meta-Analysis

**DOI:** 10.3390/diagnostics13040584

**Published:** 2023-02-05

**Authors:** I-Shiang Tzeng, Po-Chun Hsieh, Wen-Lin Su, Tsung-Han Hsieh, Sheng-Chang Chang

**Affiliations:** 1 Department of Research, Taipei Tzu Chi Hospital, Buddhist Tzu Chi Medical Foundation, New Taipei City 23142, Taiwan; 2 Department of Chinese Medicine, Taipei Tzu Chi Hospital, Buddhist Tzu Chi Medical Foundation, New Taipei City 23142, Taiwan; 3 Division of Pulmonary Medicine, Taipei Tzu Chi Hospital, Buddhist Tzu Chi Medical Foundation, New Taipei City 23142, Taiwan; 4 Department of Medical Imaging, Taipei Tzu Chi Hospital, Buddhist Tzu Chi Medical Foundation, New Taipei City 23142, Taiwan

**Keywords:** artificial intelligence, chest X-ray, SARS-CoV-2, COVID-19, summary receiver operating characteristic curve

## Abstract

Because it is an accessible and routine image test, medical personnel commonly use a chest X-ray for COVID-19 infections. Artificial intelligence (AI) is now widely applied to improve the precision of routine image tests. Hence, we investigated the clinical merit of the chest X-ray to detect COVID-19 when assisted by AI. We used PubMed, Cochrane Library, MedRxiv, ArXiv, and Embase to search for relevant research published between 1 January 2020 and 30 May 2022. We collected essays that dissected AI-based measures used for patients diagnosed with COVID-19 and excluded research lacking measurements using relevant parameters (i.e., sensitivity, specificity, and area under curve). Two independent researchers summarized the information, and discords were eliminated by consensus. A random effects model was used to calculate the pooled sensitivities and specificities. The sensitivity of the included research studies was enhanced by eliminating research with possible heterogeneity. A summary receiver operating characteristic curve (SROC) was generated to investigate the diagnostic value for detecting COVID-19 patients. Nine studies were recruited in this analysis, including 39,603 subjects. The pooled sensitivity and specificity were estimated as 0.9472 (*p* = 0.0338, 95% CI 0.9009–0.9959) and 0.9610 (*p* < 0.0001, 95% CI 0.9428–0.9795), respectively. The area under the SROC was 0.98 (95% CI 0.94–1.00). The heterogeneity of diagnostic odds ratio was presented in the recruited studies (I^2^ = 36.212, *p* = 0.129). The AI-assisted chest X-ray scan for COVID-19 detection offered excellent diagnostic potential and broader application.

## 1. Introduction

COVID-19 is a deadly pathogenic disease that results from the dissemination of coronavirus infection [1]. Acute respiratory distress syndrome, nervous system problems, organ dysfunction, or death may be caused by COVID-19 [2,3]. Thus, early recognition and prompt medical treatment of COVID-19 has become a major issue. To identify COVID-19 easily and efficiently, and to determine the prognosis, researchers have focused on researching and developing new detection methods [4]. To date, the chest X-ray is cheaper than other specialized methods. The chest X-ray is assessable from the image and has been refined during the last decade [5]. Recent evidence has indicated that the chest X-ray is a potent way of forecasting pulmonary diseases [6], respiratory diseases [7], cardiovascular diseases [8], or acute internal bleeding [9]. Cytokine storms and innate immune system overworking may trigger Acute Lung Injury (ALI) and the induction of acute respiratory distress syndrome (ARDS) related to the COVID-19 patients involved with hypertension [10]. Multiphase fibrosis, tissue stiffness, and lung function damage [11] may be caused by the hyaluronic acid (HA) molecules’ product in lung tissue, which is triggered by the cytokine storm. SARS-CoV-2 transparent cells relying on binding to the spike (S) glycoprotein of the Angiotensin-Converting Enzyme 2 (ACE2) receptor [12,13].

Therefore, a chest X-ray or computed tomography (CT) scan are recommended as first-line diagnostic tools for pulmonary involved patients [14]. Multilobar bilateral and unilateral chest X-ray, ground-glass opacity (GGO), and peripheral infiltrates on chest CT scans have been clinically proven to have a radiological role in the diagnosis of the COVID-19 disease [15,16]. For the peripheral regions of tissue, more than one lobe was found in the form of GGOs, or less nodules were found in each lobe [14,17]. The diagnosis of vascular nodules in the images of patients was difficult to attribute to the removal and large number of lung CT images and their complex and heterogeneous structures [18]. Thus, the artificial intelligence (AI) systems that assist medical imaging for screening have gained an important role in supporting decision making [19]. The AI presented an ability to change clinical decision-making; however, we should be cautious about implementing AI systems in each information system [20]. In terms of the merit of AI systems for physicians, AI systems assist as a diagnostic tool, making faster and efficient decisions. The augment ability on medical imaging in disease diagnosis is driving advances in traditional image processing and AI algorithms to retrieve diagnostic information. When diseases occur, AI provides a physician with the necessary diagnostic information required to speed up diagnosis and add precision intervention decisions. Some traditional image-assisted techniques for AI diagnosis contain contours and region progression, which provide a physician an aid with which to extract diagnostic information. Moreover, traditional models experience limited performance, customizability, and a strong reliance on in-advance computed features. AI has the ability to avoid the above limitations and to derive complex image features by importing feature semantics into classifiers [21,22,23]. In previous studies that evaluated the relationship between the use of a chest X-ray and COVID-19 incidence and death rates, it was found that the chest X-ray has not only diagnostic value but also great potential as a prediction image for clinical outcomes [24,25].

Recently, a study conducted a literature review on the diagnostic role of AI, which suggested its excellent potential and wide application relative to comparative methods, based on sufficiently sized datasets and independent testing [26]. The study found that radiographic diagnostics have sharper sensitivity than laboratory testing when compared to the numerous diagnoses of COVID-19 under development that employ AI to swiftly assess chest CT imaging [26]. Nonetheless, this study was based on a restricted review without considering a summary sensitivity and the specificity of the receiver operating characteristic curve (ROC). Hence, a more rigid multi-center study investigating the predictive role of AI-assisted chest X-ray scans for COVID-19 is guaranteed to improve our understanding of the accuracy of AI diagnostic devices.

According to the preceding study, we implemented a meta-analysis to evaluate the diagnostic value of AI-assisted chest X-ray scans to determine their accuracy in COVID-19 patients.

## 2. Materials and Methods

### 2.1. Seek Tactics and Picking Standard

We used PubMed, Cochrane Library, MedRxiv, ArXiv, and Embase to search for research published from 1 January 2020 to 30 May 2022 involving “machine learning”, “artificial intelligence”, “medical image”, “SARS-CoV-2”, and “COVID-19”, because publication related to AI was distinct from traditional therapeutic publication. Only research that considered chest X-rays to probe the usage of AI were chosen for the review. From the selected papers, the following data were excerpted: the number of datasets used for training and validation, the proportion of COVID-19 scans within the dataset, and the sensitivity, specificity, and area under curve (AUC) of the proffered manner. We also considered whether the datasets and model code were estimable. The research was then classified by imaging process: chest X-ray.

The following MeSH terms and their combinations were searched, including (Machine learning OR Artificial intelligence OR Medical image) and (SARS-CoV-2 OR COVID-19) and (AUC OR ROC OR Sensitivity OR Specificity).

### 2.2. Procedures

This review was performed in accordance with the preferred reporting items for systematic reviews and meta-analyses (PRISMA). Two researchers (IST and PCH) independently extracted the data from the included studies. Two researchers (IST and PCH) performed the initial screening, manually searching the results and selecting articles for full-text retrieval in the title (or abstract) review process. The opinion of the third reviewer (WLS) was considered if identification was inconsistent between IST and PCH. They determined eligibility by screening the titles and abstracts of the retrieved studies and extracted data by constructing a excel table. They validated any discrepancies and addressed concerns through discussion to achieve consensus in the extracted data. Information bias may be generated from an image data source, which also influenced the results of study. Several sources of imaging data included the type of imaging contexts (i.e., three- or two-dimensional), type of AI approach, sensitivity, and specificity extracted from recruited studies. The risk of bias was frontally evaluated by two independent reviewers (IST and PCH). Next, we reviewed each study using the Quality Assessment of Diagnostic Accuracy Studies (QUADAS-2) guidelines [27]. The QUADAS-2 tool was used to assess the methodological quality of the included studies [27]. The QUADAS-2 tool consisted of four key domains covering patient selection, index test, reference standard, and flow and timing. We identified the risk of bias as ’high’, ‘low’, or ‘unclear’. The result of the risk of bias for the recruited studies was presented using a plot.

### 2.3. Statistical Analysis

According to the patients with or without a COVID-19 diagnosis, we calculated counts of true positives (i.e., sensitivity multiply number of COVID-19 diagnoses), false positives (i.e., (1-specificity) multiply number of non-COVID-19 diagnoses), true negatives (i.e., specificity multiply number of non-COVID-19 diagnoses), and false negatives (i.e., (1-sensitivity) multiply number of COVID-19 diagnoses) of COVID-19 from the included research and calculated the pooled estimates for sensitivity and specificity and corresponding 95% CI.

The “mada” package [28] was used to assess the data to explore the pooled sensitivity and specificity and their 95% CI. The “mada” package [28] was also used to evaluate the summary receiver operating characteristic curve (SROC), which was used to calculate the AUC value. Finally, funnel plots were created to examine the publication bias of the included studies. All statistical analyses and graphical presentations mentioned above were implemented using R software (4.2.0 version, Vienna, Austria).

## 3. Results

### 3.1. Literature Selection and Quality Assessment

**Figure 1:**
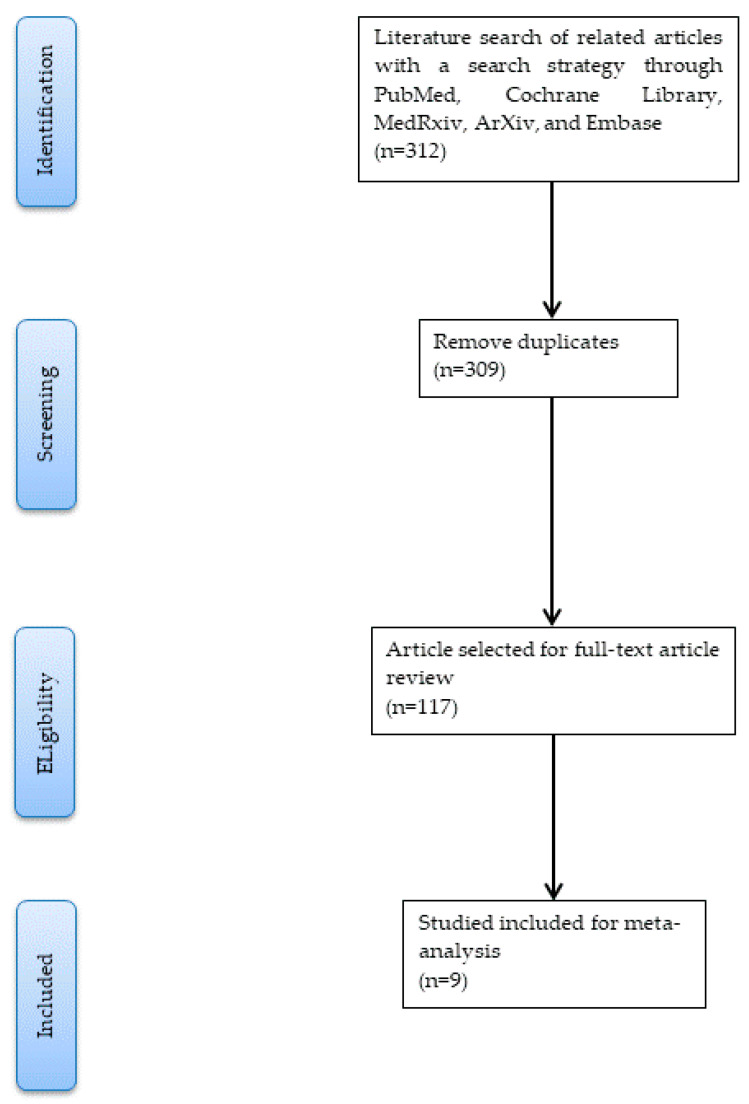
The PRISMA flowchart of study selection.

After evaluating and carefully screening all the studies from the databases, the literature search yielded nine eligible studies [29,30,31,32,33,34,35,36,37], including 39,603 participants (included 2976 COVID-19 cases and 36,627 non-COVID-19 individuals). The PRISMA flowchart of study selection is shown in Figure 1.

### 3.2. Risk of Bias Assessment

**Figure 2:**
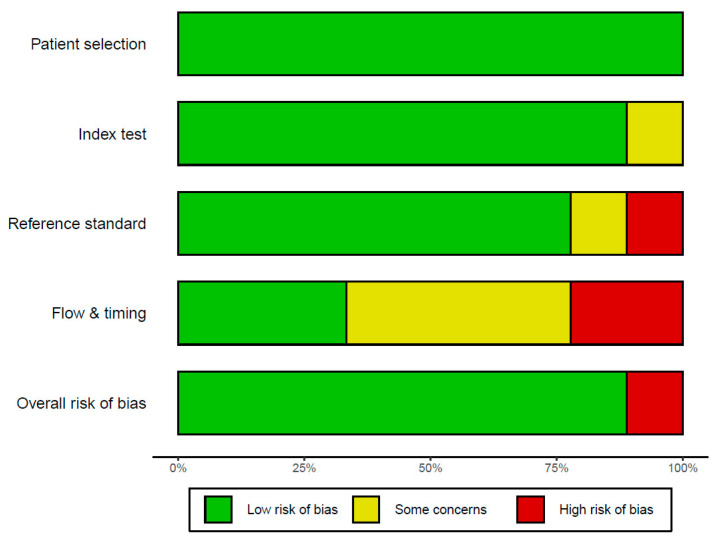
The risk of bias plot.

Based on the definition of the criterion standard for the detection of COVID-19 using chest X-rays assisted by AI system, data were extracted from the recruited studies for this meta-analysis. Extraction data included the first author, year of publication, the conducted study country, type of study, and number of patients. The QUADAS-2 tool was used to assess the quality and potential bias of nine studies. Four key domains covering patient selection, index test, reference standard, and flow and timing were assessed. The results of the literature quality assessment using QUADAS [27] are provided in Figure 2. Low risk bias implied confidence on the part of the literature reviewer that results represent the true diagnostic effect (such as sensitivity, specificity, and AUC). Figure 2 showed that 90% of the “overall risk of bias” item presented as having a low risk of bias. 

### 3.3. General Characteristics

**Figure 3:**
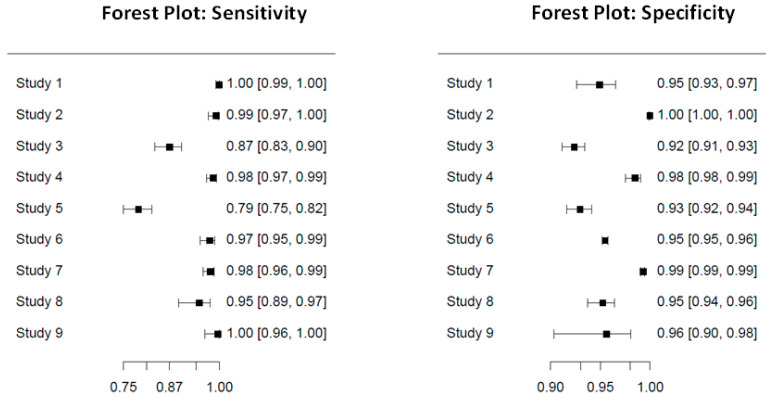
The forest plot of sensitivity and specificity results of the nine studies.

We summarize the general characteristics of all nine studies included in this meta-analysis in Table 1 and their research findings. The general characteristics of the recruited studies are shown in Table 1. We found that four studies were conducted in the United States of America and that the other five studies were conducted in different countries, respectively. Next, we included seven case-control studies and two retrospective studies. For participants in this meta-analysis, this study included 2976 COVID-19 cases and 36,627 non-COVID-19-diagnosed individuals. The sensitivity and specificity of the nine studies are presented as a forest plot in Figure 3. We also presented 95% CI of sensitivity and specificity for the nine studies in Figure 3.

### 3.4. Results of Pooled Estimates for Sensitivity and False Positive Rate Analysis

**Figure 4:**
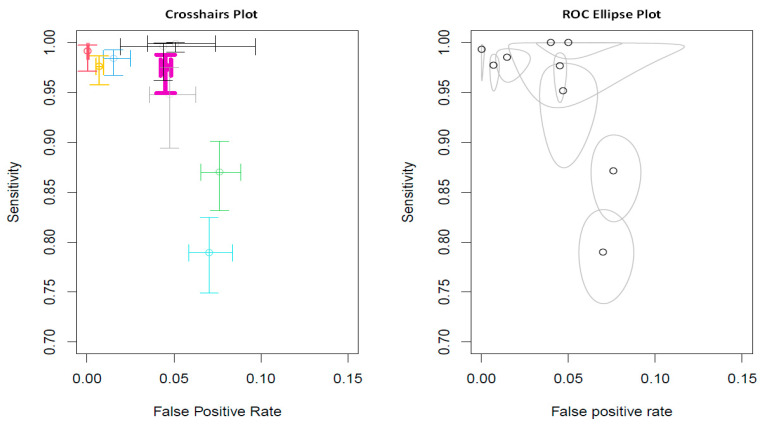
Pooled estimates for sensitivity and false positive rate analysis. Crosshairs wider with increased weight and colored arbitrarily.

**Figure 5:**
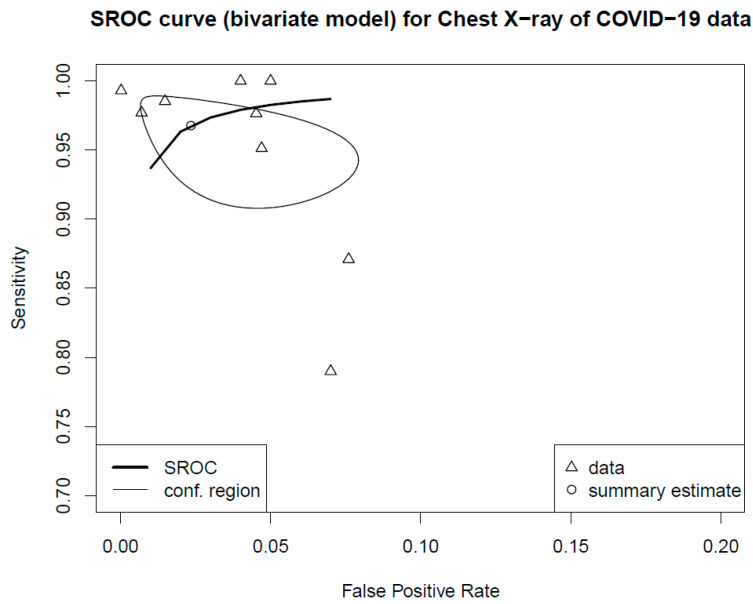
The summary operating characteristic curve and the AUC were 0.98 (95% CI 0.94–1.00). By default, the point estimate of the pair of sensitivity and false positive rate is also plotted together with a confidence region (i.e., conf.region).

For the random effects model, the pooled sensitivity was 0.9472 (*p* = 0.0338, 95% CI 0.9009–0.9959), and the pooled specificity was 0.9610 (*p* < 0.0001, 95% CI 0.9428–0.9795). Two-dimensional plots were provided by the “mada” package [28]. One is the crosshair plot, and the other is the ROC ellipse plot. In Figure 4, the crosshair was conducted using an arbitrary color, which made the crosshairs wider with increased weight. Bold purple crosshairs were presented due to their biggest weight among nine studies. The SROC was generated to assess the diagnostic value, and the results revealed an AUC of 0.98 (95% CI 0.94–1.00 in Figure 5).

### 3.5. Deeks’ Test

We used the formulas provided by Deeks [38]. The “mada” module also performed χ2 tests to evaluate the heterogeneity of sensitivities and specificities. The null hypotheses in both cases’ heterogeneity of sensitivities and specificities were equivalent. Test results for the equality of sensitivities: X-squared = 297.3168 with *p* < 0.0001; test results for equality of specificities: X-squared = 581.6488 with *p* < 0.0001. This showed that the heterogeneity results of sensitivities and specificities were not equal in both cases.

## 4. Discussion

COVID-19 is a severe acute respiratory syndrome caused by the SARS-CoV-2 virus, resulting in organ exhaustion and gradual death [39]. In September 2022, COVID-19 infections and deaths worldwide reached 620 and 6.5 million, respectively, (https://www.worldometers.info/coronavirus/, accessed on 31 January 2023). This study successfully presented overwhelming evidence suggesting that the adoption of AI-based chest X-rays is a useful diagnostic tool to detect COVID-19.

There were seven case-control studies and two retrospective studies recruited in this meta-analysis due to the search strategy adopted in this study. Moreover, we focused on diagnostics of the COVID-19 epidemic rather than the novelties of AI, as clinical research studies are more widespread for other AI utilizations. Finding accessible small datasets in the public domain is a general challenge for overall research. Because COVID-19 has occurred only since late 2019, there are few images of COVID-19 patients accessible at separate institutions and in public domain datasets. Some studies have adopted similar datasets (Table 1). This is a drawback, as the algorithm trained on a specific dataset may not have the ability to execute as well when lending itself to the assorted data [40]. Simultaneously, the scarcity of external checks among the investigated studies may enhance this risk of bias.

Our evaluation of the repeatability of the results of the algorithms may have been limited owing to smaller datasets. Nevertheless, some research used datasets in public domain but neglected their clarity regarding the origin and disposition of the image data. This may cause a certain level of bias. Another review utilizing AI to interpret COVID-19 diagnoses revealed that the high level of bias in most of their papers was an effect of the small number of COVID-19 images [37]. However, small datasets are not incompatible with research on AI in COVID-19. The stated influence may be controversial. A previous study focusing on pulmonary nodules assessed with AI was based on a small sample of 186 patients [41]. Images used in the study were found in numerous public storehouses and taken from publications [36]. These images probably presented extreme and provocative cases of COVID-19 that may have been easier for the algorithm to perceive. Furthermore, some datasets were branched through vision ascent and the development of repetition. Among the nine studies, only two studies presented an independent examination with an external dataset (22%), and the two studies that adopted external validation presented average sensitivity and specificity results of 97.56% and 95.15%, respectively. The remaining seven studies without external validation presented average sensitivity and specificity values of 94.18% and 96.38%, respectively. Thus, evaluating the performance of externally checked models was superior to disregarding externally checked models [42]. A previous study revealed in another current review of this objective [43] that only 21% (13/62) assessed their algorithms on separate datasets. High performance in external testing provided solid evidence that the model may be extrapolated to another patient population. Such external validation may mitigate influence and controversy. To date, the performance in external testing confirmed that the model could be converted into clinical practice.

Some studies constructed on the sampling from large COVID-19 datasets to mitigate the uncertainty of prediction [44,45,46,47]. Then, a previous systematic review described AI-based diagnostic imaging (CT and chest X-ray) tools and showed that the performance of both CT and chest X-ray diagnostic tools may be limited by the scale of the dataset [48]. In addition, the well-balanced data had the benefit of the training of neural networks. Minor differences of in the variable class of data significantly affect the results of study [49]. Equal classes of data are also found in a previous review [50]. However, a small number of chest X-rays are still to be found in some studies conducted throughout the pandemic [51,52,53].

According to the diagnostic ability of CT, another meta-analysis conducted based on CT imaging settled as the future work. This meta-analysis extracted the diagnostic performance of AI-assisted CT-Scan for COVID-19. Some studies which based their meta-analysis on AI-assisted CT-Scan diagnostic performance for COVID-19 demonstrated an AUC from 0.95 to 0.97 [54,55]. Consequently, the AI-assisted CT-Scan for COVID-19 was associated with pneumonia based on objective investigation [56,57]. A review of recent publications proved that physicians, in detecting COVID-19, used an AI supportive system [26]. Community acquired pneumonia and lung diseases were detected with an AUC value of 0.96 using a deep learning model [58]. On the contrary, some published studies showed the fair performance (AUC values ranged from 0.732 to 0.87) of DL model-assisted COVID-19 detection [59,60,61]. However, molecular diagnostic tests, such as reverse transcription–polymerase chain reaction (RT-PCR), were still the most reliable diagnostic tool, rather than the AI-assisted CT-Scan for COVID-19 detection [62]. Because lungs are infected at the later stage of infection and present a certain level of confusion in identification through medical imaging (Figure S1), the importance of RT-PCR tests cannot be underestimated. Controversially, AI, which assisted the chest CT-Scan to classify diseases’ status, has an excellent performance of diagnoses, with AUC values ranging from 0.90 to 1.00 [36,63,64,65]. 

This study utilized the SROC analysis of diagnostic accuracy in the adoption of AI-assisted chest X-rays for the detection of COVID-19. In this meta-analysis, a strictly screened literature search was performed for all studies published in English between 1 January 2020 and 30 May 2022. The pooled estimates for sensitivity and specificity were 0.9472 (*p* = 0.0338, 95% CI 0.9009–0.9959) and 0.9610 (*p* < 0.0001, 95% CI 0.9428–0.9795), respectively. To evaluate the diagnostic value and prediction accuracy of using AI-assisted chest X-rays for COVID-19, the SROC curve indicated that the AUC value was 0.98 (95% CI 0.94–1.00). The results of this study suggested that the use of AI in chest X-rays has a significant value for forecasting COVID-19. Soda et al. [66] determined that the AUC of AI in chest X-rays in predicting COVID-19 was 0.63 (95% CI 0.52–0.74); nevertheless, only older adults aged above 65 years were included, and the number of subjects was not large. Another review study [67] summarized the literature of AI in chest X-rays for predicting COVID-19, albeit without synthesis evidence. 

This meta-analysis verified the prediction value of AI in chest X-rays for COVID-19 patients (Table 2); however, there were some limitations and shortcomings. First, there was notable but not significant heterogeneity (I^2^ = 36.212, *p* = 0.129) on the diagnostic odds ratio in the review; the random effect results of the pooled sensitivity and specificity were 94.72 (*p* = 0.0338, 95% CI 90.09–99.59) and 96.10 (*p* < 0.0001, 95% CI 94.28–97.95), respectively. The funnel plot of the sensitivity and specificity may show a certain selection bias for recruited studies (Figure S2). However, such heterogeneity may have resulted from dissimilarity in areas, the cut-off value of AI in chest X-rays, the severity of COVID-19, and the resolution of the chest X-ray (Figure S2). Moreover, the predictive value of AI in chest X-rays was not consistent, and the predictive value of AI in chest X-rays had not been considered in the preceding studies, which may have negatively influenced the contributed sensitivity and specificity. Deep learning is a type of machine learning. Moreover, the goal of machine learning is to enable computers to think and behave independently of human input. However, deep learning is the process of teaching computers to reason by using structures inspired by the human brain. The recruited nine studies, which focus on a deep learning model (Table 1), may limit the results of this study.

## 5. Conclusions

In conclusion, AI can play a significant auxiliary role in utilizing chest X-rays when diagnosing COVID-19. Considering that COVID-19 is a complicated syndrome involving complex pathophysiological mechanisms, AI can assist chest X-rays though should not be considered as the single decisive signal for perceiving COVID-19. Other elements such as medical history, physical examination, and pathogenic microorganism tests should be implemented during the clinical diagnostic procedure. Future studies should consider clinical comparisons and external validation.

## Figures and Tables

**Table 1:** The general characteristics of the recruited studies.

Study Number	First Author	Publication Year	Country	Study Type	Dataset	Deep Learning Model	All Data	COVID	Non-COVID	Sensitivity	Specificity
1	Borkowski [29]	2020	United States of America	Case control	COVID-19/non- COVID pneumonia/ COVID-19/non-COVID pneumonia/normal	Microsoft CustomVision	1000	500	500	100	95
2	Zokaeinikoo [30]	2021	United States of America	Case control	COVID-19/non- COVID infection/ normal	AIDCOV using VGG-16	5801	269	5532	99.3	99.98
3	Keidar [31]	2021	Israel	Retrospective	COVID- 19/normal	RetNet50	2427	360	2067	87.1	92.4
4	Ahmed [32]	2021	Japan	Case control	COVID/non- COVID	HRNet	1410	410	1000	98.53	98.52
5	Kikkisetti [33]	2020	United States of America	Retrospective	COVID/bacterial pneumonia/ viral pneumonia/ normal	VGG-16	2031	445	1586	79	93
6	Shibly [34]	2020	Bangladesh	Case control	COVID/non- COVID	Faster R-CNN	19,250	283	18,967	97.65	95.48
7	Gomes [35]	2020	Brazil	Case control	COVID- 19/bacterial and viral pneumonia	IKONOS	6320	464	5856	97.7	99.3
8	Ko [36]	2020	South Korea	Case Control	COVID- 19/pneumonia/ normal	DarkNet-19	1125	125	1000	95.13	95.3
9	Sharma [37]	2020	United States of America	Case control	COVID-19/non COVID-19	Residual Att Net	239	120	119	100	96

**Table 2:** The characteristics and performance of diagnostic test results.

		Test Result	
	Total (*n* = 39,603)	Positive	Negative
True condition	COVID-19 (*n* = 2976)	2804.79	171.21
	Non COVID-19 (*n* = 36,627)	1259.0788	35,367.9212

## Data Availability

The data used to support the findings of this study are including within the article.

## Supplementary Materials

The following supporting information can be downloaded at: https://www.mdpi.com/article/10.3390/diagnostics13040584/s1, Figure S1: Funnel plots for recruited 9 articles according to sensitivity and specificity. Figure S2: Medical images of COVID-19, pneumonia patients, and normal individual.
