# Supplementary material for: Artificial Intelligence-Assisted Chest X-ray for the Diagnosis of COVID-19: A Systematic Review and Meta-Analysis
Source: Diagnostics (Basel). 2023 Feb 5;13(4):584. doi: 10.3390/diagnostics13040584 (PMC9955250; doi:10.3390/diagnostics13040584)
Supplement: Supplementary file 1 [file diagnostics-13-00584-s001.zip › Figure_S1.pdf]

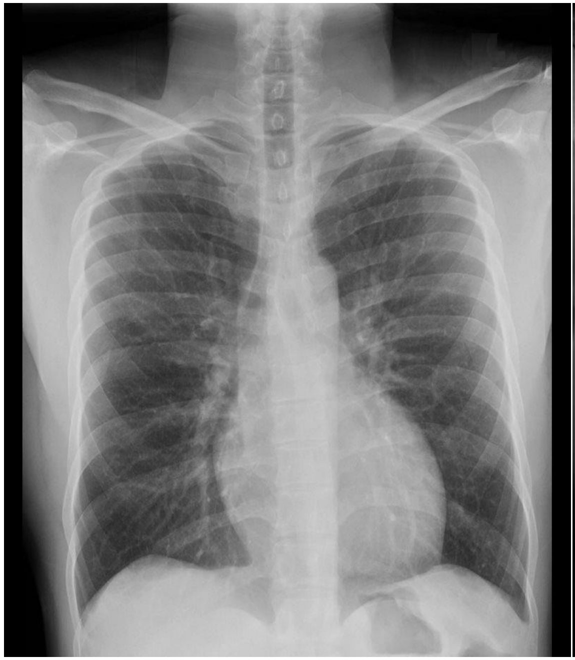

Normal

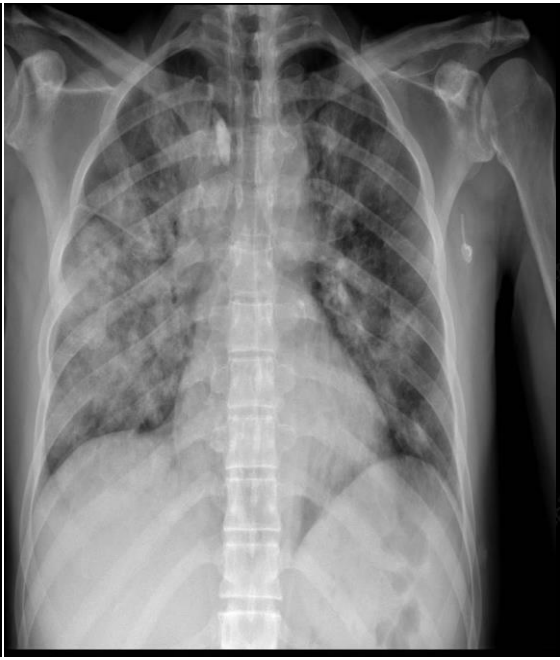

COVID-19

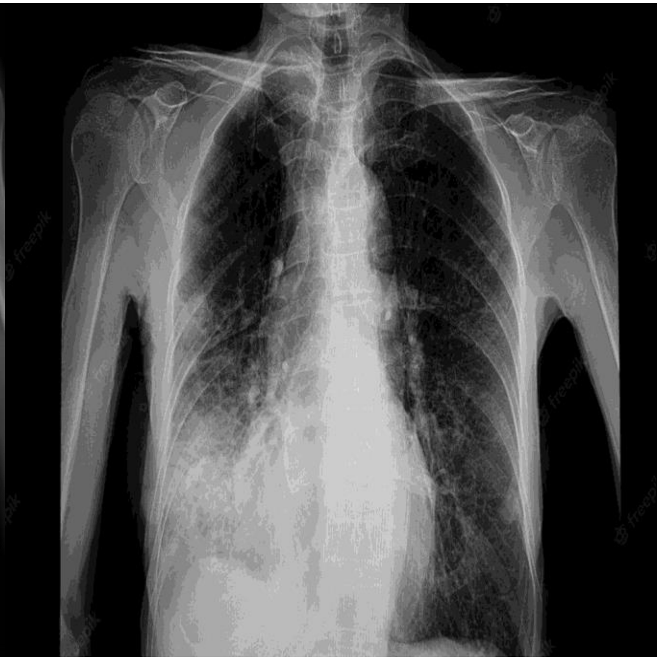

Pneumonia (right lower lung)

Source: https://www.kaggle.com/datasets/alifrahman/covid19-chest-xray-image-dataset;
https://lungs.thecommonvein.net/chest-x-ray-normal-cxr/;
https://www.freepik.com/premium-photo/pneumonia-right-lower-lung_5064784.htm
