# Supplementary figures and images for: Artificial Intelligence-Assisted Chest X-ray for the Diagnosis of COVID-19: A Systematic Review and Meta-Analysis
Source: Diagnostics (Basel). 2023 Feb 5;13(4):584. doi: 10.3390/diagnostics13040584 (PMC9955250; doi:10.3390/diagnostics13040584)

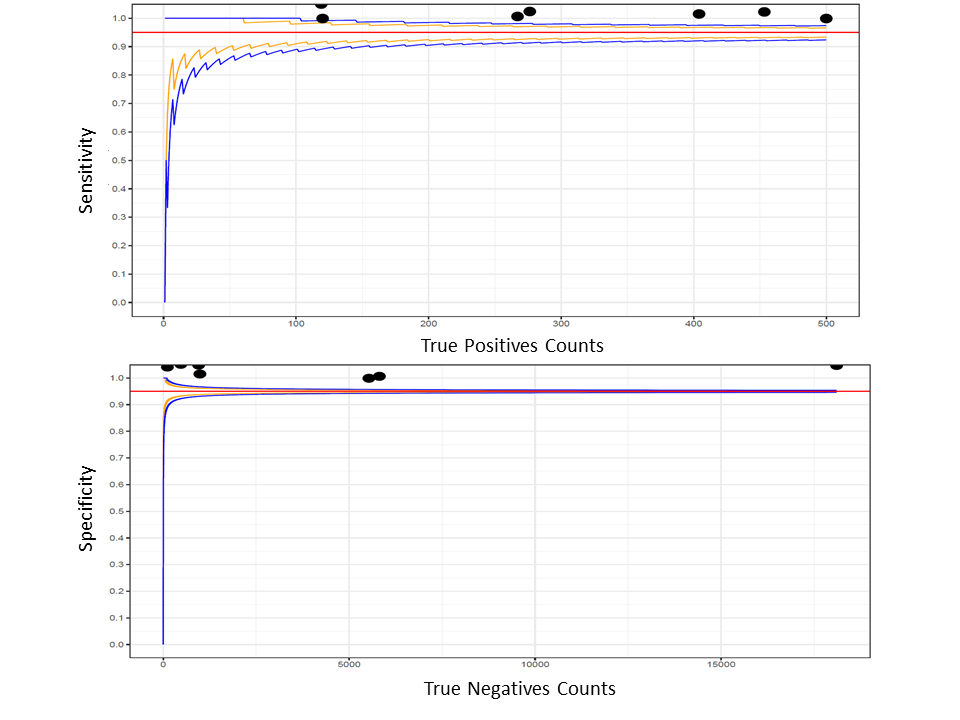

Supplement: Supplementary file 1 [file diagnostics-13-00584-s001.zip › Figure_S2.tif]
